# Supplementary material for: Pro-Fibrotic Effects of CCL18 on Human Lung Fibroblasts Are Mediated via CCR6
Source: Cells. 2024 Jan 26;13(3):238. doi: 10.3390/cells13030238 (PMC10854834; doi:10.3390/cells13030238)
Supplement: Supplementary file 1 [file cells-13-00238-s001.zip › cells-2777986-SI.pdf]

Supplementary Data and Methods

Extra- and intracellular flow cytometry

Fibroblasts were harvested from the culture flasks by gentle trypsinization. As CCR6 is a trypsin-sensitive molecule, trypsinized cells were allowed to recover in medium in polystyrene tubes (to avoid adhesion) for at least 3h at 37°C before staining. After this period, cells were washed again in PBS and fixed using 200µl paraform-aldehyde (4%, Sigma, Taufkirchen, Germany) for 5 minutes on ice. The reaction was stopped by the addition of PBS containing 1% FCS and human non-labelled IgG. Surface expression of CCR6 was stained using a FITC-labelled anti-human CCR6 antibody (R&D Systems, Minneapolis, MN) and a suitable IgG control for 20 minutes. Cells were then washed with PBS/FCS/hIgG and permeabilized using PBS/FCS/hIgG containing 0.1% saponin (Sigma, Sigma, Taufkirchen, Germany). Intracellular expression of CCR& was determined using the same antibody as above in presence of 0.1% saponin for 20 minutes. The cells were then washed twice using PBS/FCS/hIgG/saponin. Fibroblasts populations were gated by forward/sideward scatter and the expression of CCR6 was estimated in comparison with the appropriate control IgG. The results are depicted in Fig. S5

Induction of CCR6 expression on fibroblasts

Fibroblasts were seeded at a density of 300.000 cells per well in a 6-well cell culture plate in Quantum 333 medium. Cells were allowed to attach overnight and the medium was replaced with 1 mL of DMEM plus 10% FCS. Cells were either left non-stimulated or were stimulated with IL-4 (10 ng/mL), IL-13 (10 ng/mL), IL-10 (10 ng/mL) or TGFβ (5 ng/mL) and cultured for 24h. Cells were then removed using trypsin and stained for CCR6 expression as described in Material and Methods.

Fig. S1

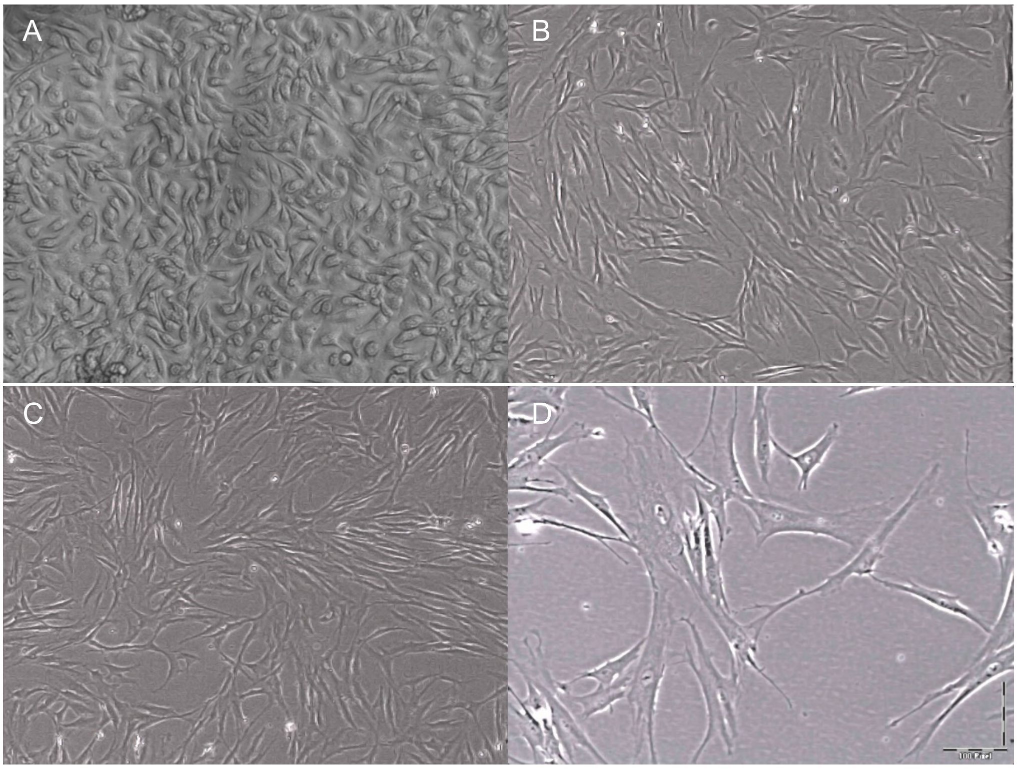

Figure S1 Phase contrast microscopy photographs from a control fibroblast line (A) and two fibroblast lines from patients suffering from IPF (A-C magnification 40-fold; D magnification 100-fold)

Fig. S2

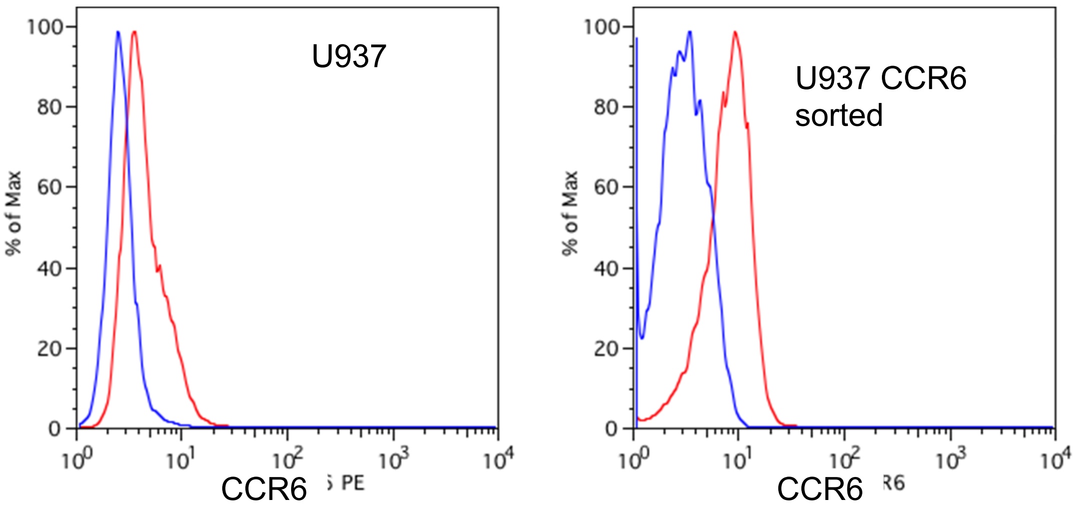

Figure S2 CCR6 expression of non-sorted and CCR6 sorted U937 cells. CCR6 sorted U937 cells express higher levels of CCR6

Fig S3

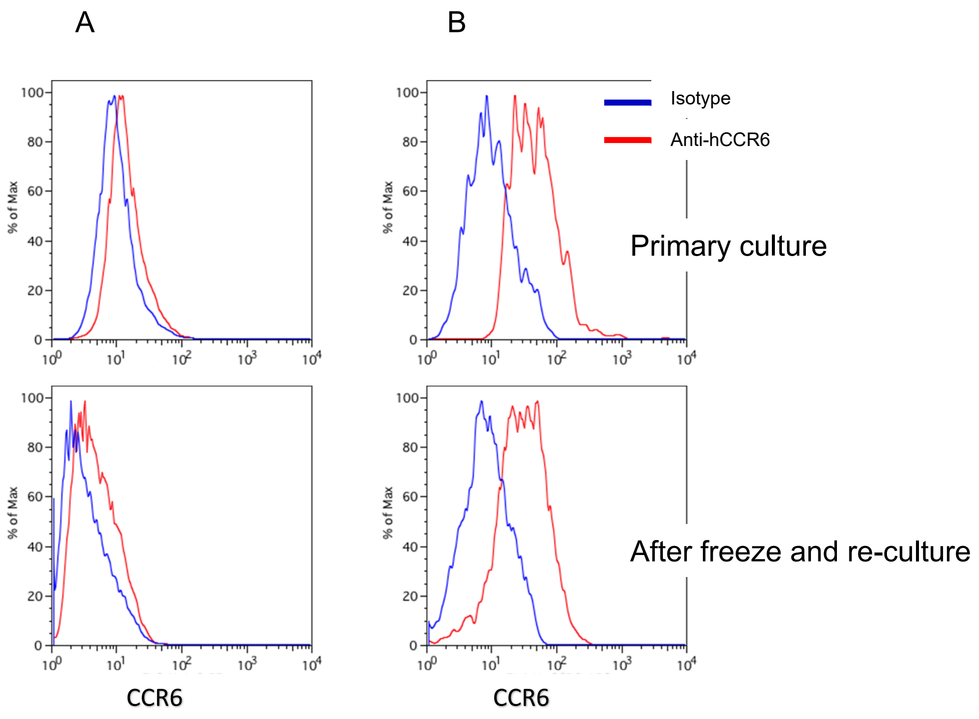

Figure S3 CCR6 expression of a low(A) and a high (B) CCR6-expressing fibroblast line at primary culture (upper panel) at the 5th and 6th passage. The cells were frozen and stored in liquid nitrogen and re-cultured after 2 and 4 years respectively equaling the 10th and 12th passage. The cells disclose only marginal differences in the CCR6 expression pattern.

Fig S4

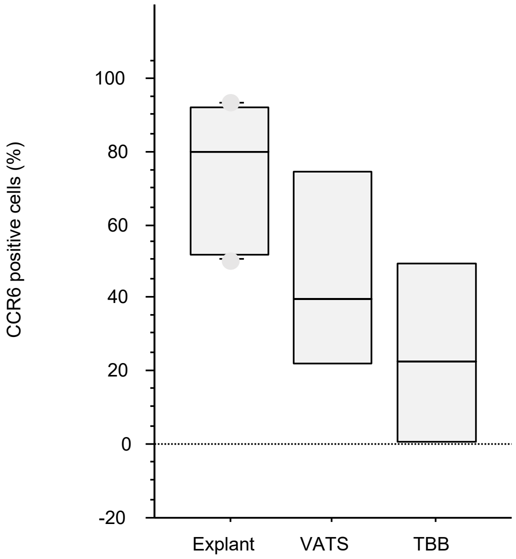

Figure S4 Expression of CCR6 on fibroblasts established from different material sources. Highest expression was seen from fibroblast lines established from explant lungs. Lowest expression was seen in lines deriving from transbronchial biopsies. The differences did not reach statistical significance.

Fig S5

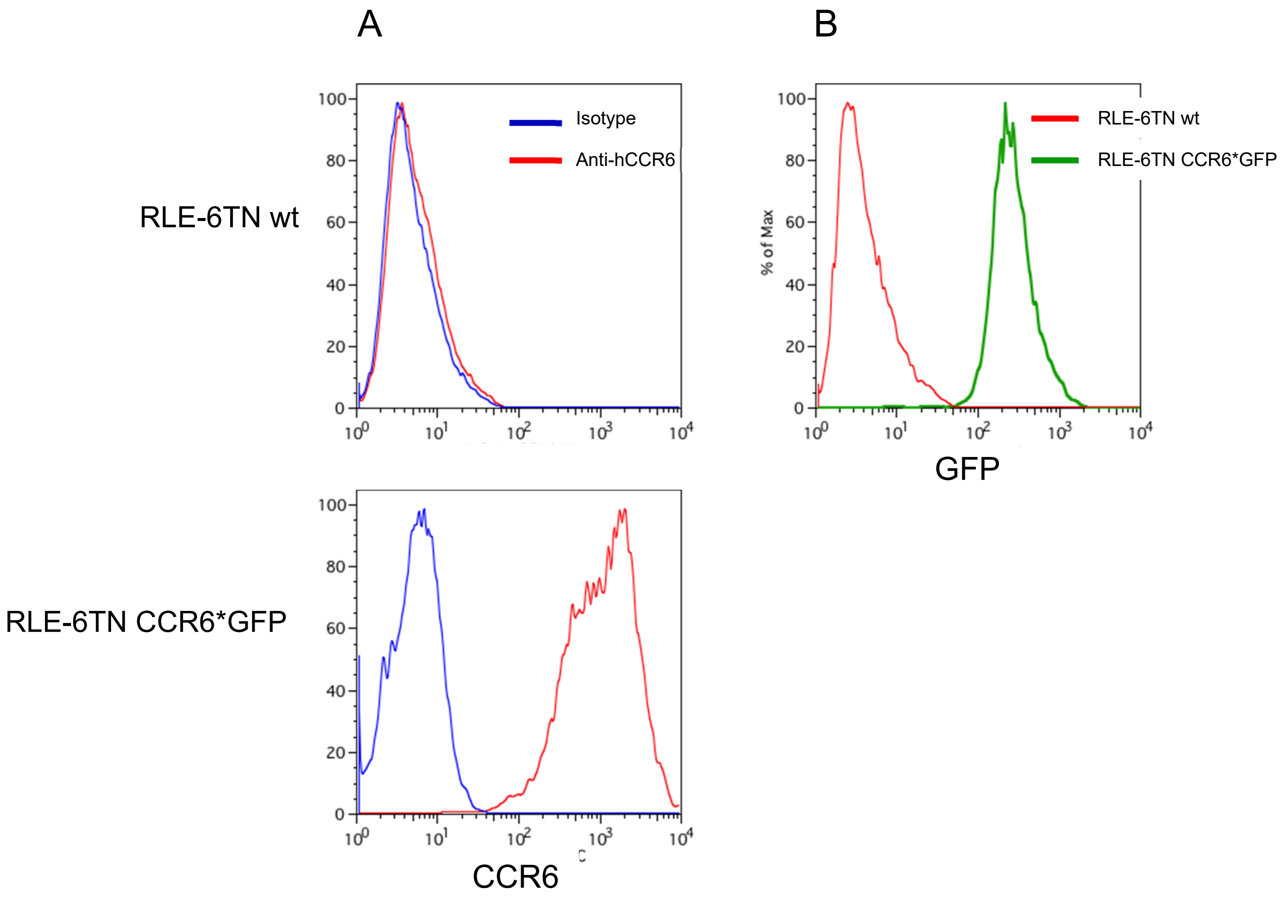

Figure S5 A) Expression of CCR6 before (upper panel, wild type) and after (lower panel) transfection of the RLE-6TN cells with the CCR6*GFP lentiviral vector. B) GFP fluorescence of the wild type RLE6TN (red) and the RLE-6TN CCR6*GFP cells (green).

Fig S6

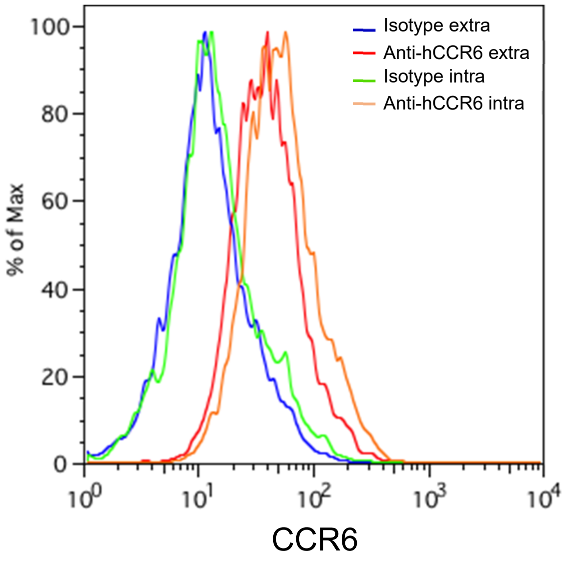

Figure S6 Detection of CCR6 expression without (red) and with (orange) cell permeabilization of a positive lung fibroblast line. Isotype controls are depicted in green and blue. A shift to increased fluorescence intensity is visible after cell permeabilization and additional CCR6 staining indicating an intracellular reservoir of CCR6.

Fig S7

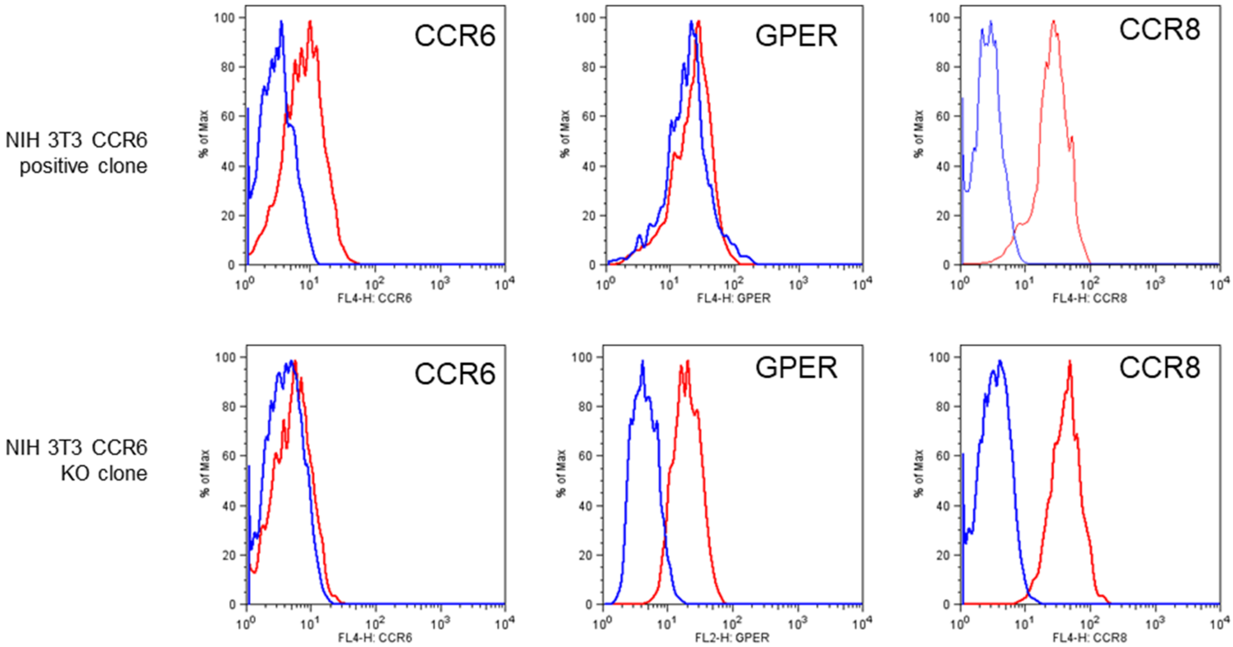

Fig. S7 The CCR6 positive NIH 3T3 clone expresses no GPER whereas the CCR6 KO clone was clearly positive. Both clones were highly positive for CCR8 and negative for PITPNM3.

Fig S8

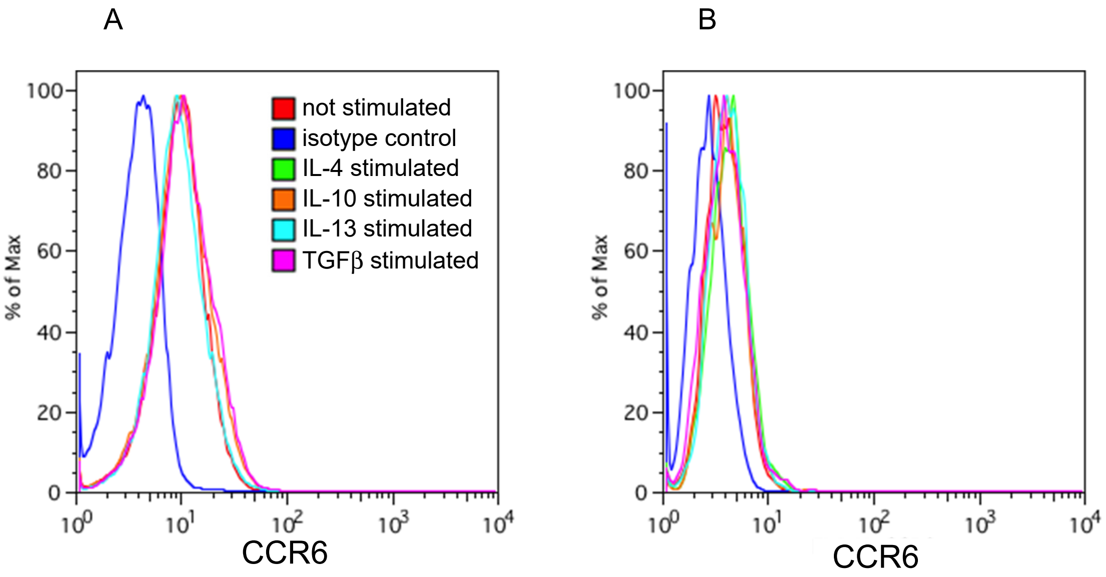

Fig. S8 Stimulation of two fibroblast lines with high (A) or low (B) CCR6 expression with IL-4 (10 ng/mL), IL-13 (10 ng/mL), IL-10 (10 ng/mL) or TGFb (5 ng/mL) for 24h did not change the expression level of CCR6.
